# Study of Radiation Characteristics of Intrinsic Josephson Junction Terahertz Emitters with Different Thickness of Bi_2_Sr_2_CaCu_2_O_8+δ_ Crystals

**DOI:** 10.3390/ma14051135

**Published:** 2021-02-28

**Authors:** Takanari Kashiwagi, Takumi Yuasa, Genki Kuwano, Takashi Yamamoto, Manabu Tsujimoto, Hidetoshi Minami, Kazuo Kadowaki

**Affiliations:** 1 Graduate School of Pure & Applied Sciences, University of Tsukuba, 1-1-1 Tennodai, Tsukuba 305-8571, Japan; taku1511tkb@gmail.com (T.Y.); s-kuwano@ims.tsukuba.ac.jp (G.K.); tsujimoto@ims.tsukuba.ac.jp (M.T.); minami@bk.tsukuba.ac.jp (H.M.); 2 Division of Materials Science, Faculty of Pure & Applied Sciences, University of Tsukuba, 1-1-1 Tennodai, Tsukuba 305-8573, Japan; 3 QuTech, Delft University of Technology, PO Box 5046, 2600 GA Delft, The Netherlands; T.Yamamoto@tudelft.nl; 4 Algae Biomass and Energy System R & D Center, University of Tsukuba, 1-1-1 Tennodai, Tsukuba 305-8572, Japan; dr.kazuo.kadowaki@gmail.com

**Keywords:** intrinsic Josephson junctions, high temperature superconductors, terahertz waves

## Abstract

The radiation intensity from the intrinsic Josephson junction high-$T_{c}$ superconductor Bi${}_{2}$Sr${}_{2}$CaCu${}_{2}$O${}_{8+\delta }$ terahertz emitters (Bi2212-THz emitters) is one of the most important characteristics for application uses of the device. In principle, it would be expected to be improved with increasing the number of intrinsic Josephson junctions *N* in the emitters. In order to further improve the device characteristics, we have developed a stand alone type of mesa structures (SAMs) of Bi2212 crystals. Here, we understood the radiation characteristics of our SAMs more deeply, after we studied the radiation characteristics from three SAMs (S1, S2, and S3) with different thicknesses. Comparing radiation characteristics of the SAMs in which the number of intrinsic Josephson junctions are *N*∼ 1300 (S1), 2300 (S2), and 3100 (S3), respectively, the radiation intensity, frequency as well as the characteristics of the device working bath temperature are well understood. The strongest radiation of the order of few tens of microwatt was observed from the thickest SAM of S3. We discussed this feature through the $N^{2}$-relationship and the radiation efficiency of a patch antenna. The thinner SAM of S1 can generate higher radiation frequencies than the thicker one of S3 due to the difference of the applied voltage per junctions limited by the heat-removal performance of the device structures. The observed features in this study are worthwhile designing Bi2212-THz emitters with better emission characteristics for many applications.

## 1. Introduction

High performance terahertz (THz) emitters, detectors, and the related devices have a great potential for fundamental and applied research fields in material sciences, because vibration modes for such polymers, molecules, and proteins exist in the frequencies of THz region. Stronger THz emission with coherent and monochromatic radiation has been attracted for the most of applications such as non-destructive inspections, security checking, identification of chemical substances, and cancer detection [1,2,3,4]. Recent rapid development of THz emitters and detectors has been motivated by many such useful applications [5,6].

As for THz emitters, semiconducting solid state devices such as resonant tunneling diodes (RTDs) [7,8,9,10] and quantum cascade lasers (QCLs) [11,12,13,14] have been well developed. Nowadays RTDs with sub-mW levels of output power operating at room temperature can be obtained. However, in order to generate higher output power above 1 THz, there are technical difficulties [5,7]. On the other hand, QCLs have good output characteristics, e.g., 138 mW at 4.4 THz [12] and can generate frequencies ranging from 1.2 to 5.4 THz. However, low temperature operation is required in order to obtain emission frequency around 1 THz [13]. According to the emission principle of the QCLs, the operating maximum temperatures of QCLs are restricted by $T_{\max}$(K) = (*h*/$k_{B}$)*f* ∼ 50*f* (THz), where *h* is Planck’s constant and $k_{B}$ is Boltzmann’s constant [11]. Recently, THz-QCLs using difference-frequency generation QCL techniques operating at the room temperature have been developed [6,15,16,17].

**Figure 1:**
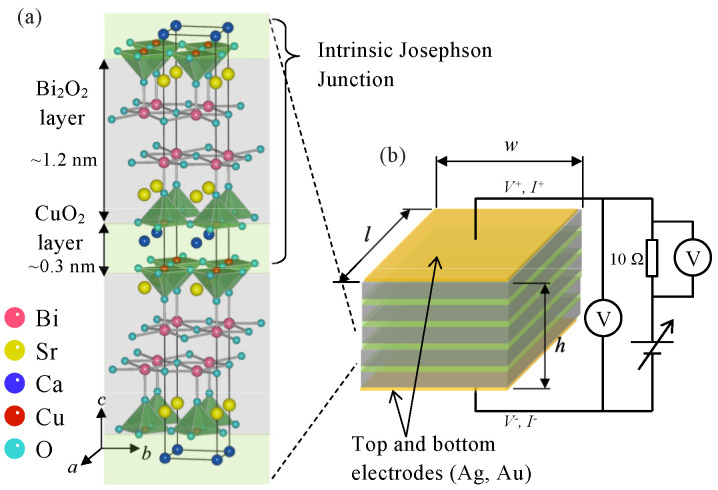
Sketch of the crystal structure of Bi2212 (**a**) and schematic picture of the mesa structure (SAM) and its wiring (**b**). It is noted that the crystal structure is drawn by VESTA [73].

THz emitters based on the ac Josephson effect have been developed by using a piece of a single crystal of the high-$T_{c}$ superconductor Bi${}_{2}$Sr${}_{2}$CaCu${}_{2}$O${}_{8+\delta }$ (abbreviated as Bi2212). It is well known that the crystal structure of Bi2212 gives rise to a regular stack of multiple Josephson junctions. Alternate layers of superconducting CuO${}_{2}$ and insulating Bi${}_{2}$O${}_{2}$ are stacked along the crystallographic *c*-axis as displayed in Figure 1a. These atomic scales of Josephson junctions with a thickness of ∼1.5 nm are known as the intrinsic Josephson junctions (abbreviated as IJJs) [18,19,20]. A mesa structure of Bi2212 single crystals displayed schematically in Figure 1b works as a THz emitter (abbreviated as Bi2212-THz emitter) by applying a dc voltage $V_{\mathrm{dc}}$ across the stack of IJJs [21]. Bi2212-THz emitters have monochromatic, coherent, and continuous sources of THz waves with a variable frequency range according to the ac Josephson effect [22,23,24,25,26,27]. It is also noted that not only IJJs but also Josephson junctions and grain boundaries in high-$T_{c}$ superconductors can be applicable to high-power and electronic device applications [28,29].

A pioneering study performed by Ozyuzer et al. [21] open a new field of THz emitting devices by using high-$T_{c}$ Bi2212 single crystals. The characteristics of the electromagnetic waves radiating from these Bi2212-THz emitters are that they are coherent, continuous, widely tunable from 0.3 to 2.4 THz [21,30,31,32], with even higher frequencies that can be generated up to 11 THz [33], their narrow spectral linewidth is less than 0.5 GHz [34], and has even been reported to be as low as 20 MHz [35], and they are highly polarized, either linearly or circularly [36,37]. Furthermore, Bi2212-THz emitters can be operated at temperatures up to 86 K [38]. To date, the maximum intensity observed from a single mesa device is about 30∼100 μW [30,39,40,41,42,43]. However, an output power of 610 μW level at 0.51 THz was recorded by cooperative parallel operation of three mesa devices [42]. Very recently, an external cavity structure is also developed in order to control the radiation frequency above 1 THz [44]. Using these THz emitters, several demonstrations of THz imaging and THz spectroscopy based on the Bi2212-THz emitters were performed [39,45,46,47,48,49]. More detailed characteristics of Bi2212-THz emitters have been reviewed in several papers [50,51,52,53,54].

According to the previous studies of Bi2212-THz emitters, it has become clear that Joule heating of the IJJs mesa structures strongly affects the emission frequencies and intensities. The experimental results obtained from local temperature measurement techniques for mesa structures of Bi2212 crystals revealed the very inhomogeneous local temperature distributions T(r) of the mesas, often called a hot-spot when the local temperature T(r) exceeds the superconducting transition temperature $T_{c}$ [55,56,57,58,59,60,61,62,63,64]. Several device structures have been proposed to overcome the heating effect [30,31,41,43,65]. Due to these improvements in the design of Bi2212-THz emitters by adding heat exhausting structures, the device characteristics such as the radiation intensities, frequencies [30,31,43], and the device working bath temperatures [38,66,67] have also improved. According to these results and the related previous studies [68,69,70,71,72], it is certain that thermal managements, such as adding heat exhaust structures, control of the number of active Josephson junctions in mesa structures, position of the hot-spot on mesa structures, and contact resistance of electrodes on mesa structures, are one of the important factors to improve the device characteristics.

In order to further develop the device characteristics of Bi2212-THz emitters, improvements in the fabrication techniques and processes of the mesa devices are required. We developed a device structure by using a stand alone type of mesa structures (abbreviated as SAMs) to reduce Joule heating of mesa structures and reported the improved radiation characteristics [30,41,43,52,65].

In this report, in order to understand the radiation characteristics of our proposed device structure of SAMs more deeply, we studied the radiation characteristics of three SAMs made of different thicknesses of Bi2212 crystals. The thickness of the SAM is related to the number of the Josephson junctions, *N*, in it and would be related to the radiation intensity, *P*. According to the previous study [21], *P* is expected to be proportional to $N^{2}$. From the comparison of radiation characteristics of these samples, the radiation intensity, the radiation frequency, and the device working bath temperature characteristics are well understood on the basis of the difference of the samples. Some of the observed results required additional research by using well size controlled SAMs; however, the results in this study are very useful to make Bi2212-THz emitters with desired emission characteristics.

In the following sections, we first explain our device structures and samples. Then the device characteristics of the SAMs such as current–voltage characteristics, radiation intensity, and frequency characteristics are described. The observed radiation characteristics of each sample are then discussed.

## 2. Materials and Methods

High-quality single crystals of Bi2212 were grown by a traveling-solvent floating-zone method using a modified infrared image furnace [74,75]. We first annealed a piece of a Bi2212 single crystal overnight to obtain an appropriate doping level [30,34,43]. Both surfaces of a freshly double-cleaved part of that crystal were coated with Ag and Au films 50 to 100 nm in total thickness by evaporation. It is known experimentally that the deposition film of Ag on Bi2212 crystals has a role to reduce a contact resistance between Bi2212 crystals and electrodes. The deposited thin film of Au on the Ag film has a role to protect oxidation of the Ag thin film. Then, rectangular SAMs, as shown schematically in Figure 1b, were fabricated by using an Ar-ion milling with metallic masks as reported in previously [43].

Three SAMs with different thicknesses denoted hereafter as S1, S2, and S3, were fabricated. The dimensions of the SAMs are *w* × *l* × *h* = 100 × 400 × 2.0 μm (S1), 80 × 405 × 3.5 μm (S2), and 58–66 × 360 × 4.8 μm (S3), respectively. Note that the thicknesses of the samples include the Ag and Au layers and these were measured using an atomic force microscope. As displayed in Figure 1a, the thickness of the single intrinsic Josephson junction is ∼1.53 nm. Bi2212 crystals with a thickness of 1 μm contain about 650 ( ≃1000 nm/1.53 nm) Josephson junctions. The total number of Josephson junctions *N* in each sample can be estimated from the thickness of the SAM. The estimated *N* of three samples are *N* ∼ 1300 (S1), 2300 (S2), and 3100 (S3), respectively. It is noted that the partial device characteristics of S3 were already reported in our previous study [52].

**Figure 2:**
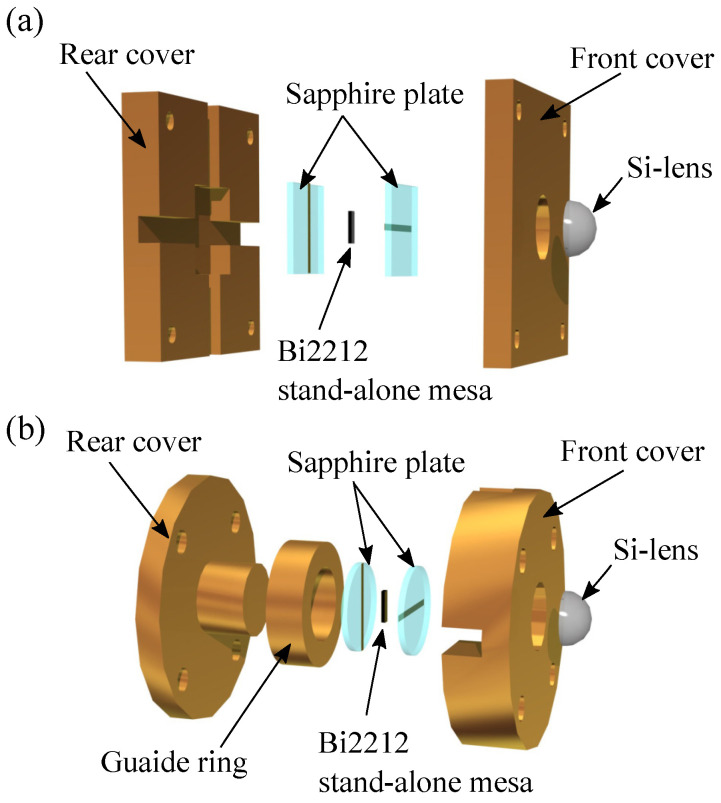
Sketch of two types of the contact-rig components used for mechanical attachment of the electrical contacts onto the SAM. Square shape (**a**) and cylindrical shape (**b**) of contact-rig components are displayed.

Figure 2 shows a sketch of two types of contact-rig components used for mechanical attachment of electrical contacts onto the SAMs. The SAMs were sandwiched by two metallic film coated sapphire substrates in order to make good electrical and thermal contacts. These rigs were fixed by four screws, then, a hemispherical-shaped Si lens with a diameter of 4 mm was attached to the top of the front sapphire substrate [43,52,65]. The electromagnetic waves from the SAMs were collected by the lens.

We have already studied the radiation characteristics of various types of SAMs by using the square shape rigs shown in Figure 2a [43,52] and the cylindrical shape rigs displayed in Figure 2b [65] and confirmed that almost similar electric and cooling properties can be obtained. These rigs enable us to make easy electrical contact to the SAMs as well as to obtain high heat exhausting characteristics from the SAMs. In this study, we used the square one for S1 and the cylindrical one for S2 and S3. It is noted that the cylindrical one was developed in order to obtain a homogeneous contact pressure to the SAMs. In the case of the square one, the contact pressure to SAMs is very sensitive to the parallelism between the rear and the front covers. This kind of difficulty can be improved by using a coaxial structure with a guide ring as seen in the cylindrical one displayed in Figure 2b.

In order to measure the radiation characteristics of the assembled SAMs, these were placed in a ${}^{4}$He-flow cryostat (OptistatCF-V, Oxford Instruments). An FeRh thermometer is placed on the sample holder inside the ${}^{4}$He-flow cryostat to measure the bath temperature $T_{b}$ of the sample. The bath temperature dependencies of the *c* axis resistance of the sample were obtained using a source meter (2400, KEITHLAY) and a temperature controller (ITC 5035, Oxford Instruments). As shown in Figure 1b, the current–voltage characteristics (IVCs) of the samples were obtained by two-wire method using a standard resistance of 10 Ω, two digital multimeters (34420A, HP), and a programmable DC voltage/current generator (R6144, ADVANTEST). For the measurement of IVCs, we selected a voltage sweeping mode of the generator. A ${}^{4}$He-cooled Si-composite bolometer (Infrared Laboratories), a lock-in-amplifier (MODEL 5210, EG&G PRINCETON APPLIED RESEARCH), and an optical chopper (300CD, Scitec instruments) were used for a lock-in detection of radiation from the emitters. The radiation frequency *f* was measured by a Fourier transform infrared (FT-IR) spectrometer with a 7.5 GHz resolution (FARIS-1, JASCO). The above experimental apparatuses were also used in our previous studies [30,34,40,41,43].

## 3. Results

**Figure 3:**
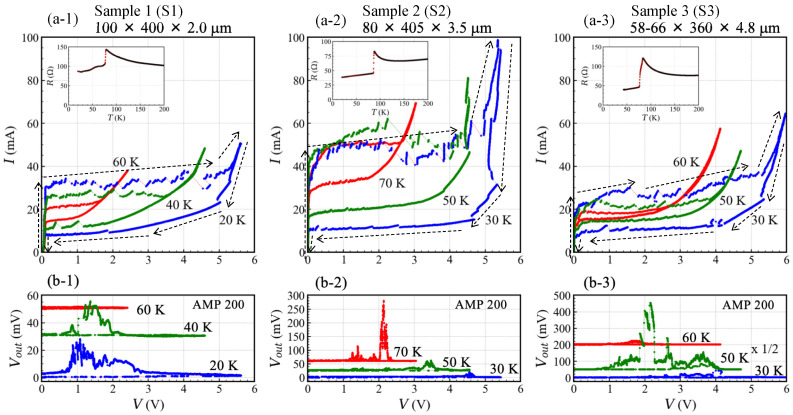
(**a**) Temperature dependence of the current–voltage characteristics (IVCs) (upper 3 panels ((**a-1**)–(**a-3**))). Inset of each IVC shows the bath temperature dependence of the *c* axis resistance. (**b**) The radiation intensity detected by the bolometer, $V_{\mathrm{out}}$, is plotted as a function of the applied voltage to the SAM (lower 3 panels ((**b-1**)–(**b-3**))). It is noted that an amplifier gain (AMP) is displayed on upper right of the figures and the $V_{\mathrm{out}}$ of sample S3 at 50 K is measured with the AMP of 100.

The bath temperature dependencies of the *c* axis resistance (RT) of the SAMs measured using the two-wire method are displayed in the inset of Figure 3a. These were obtained by applying a dc current amplitude of 0.1 mA in a $T_{b}$ increasing process. Superconducting transition temperatures of three samples estimated from the onset of the RT data are $T_{c}$ ≃ 78 K(S1), 86 K(S2), and 84 K(S3), respectively. The difference of $T_{c}$ suggests the difference of the carrier doping level of the samples. It would be originated from the difference of oxygen contents of the crystals [76]. A possible reason of a broader transition temperature (Δ$T_{c}$ ∼ 8 K) seen in the sample S3 comparing to other samples may be related to inhomogeneity of oxygen contests in the crystal.

A residual resistance below $T_{c}$ seen in the RT data can be attributed to a contact resistance between the SAMs and the electrodes, top and bottom electrodes on the SAMs made of Ag and Au, a resistance of the metallic thin films on the sapphire substrates, and a resistance of wires used in the measurement. It is noted that a four-wire method is the most proper way to evaluate applied bias voltages to Josephson junctions because some of the resistances behave like a resistance connected to the Josephson junctions in series.

As seen in the RT data of S2 and S3, the residual resistance below $T_{c}$ decease linearly with decreasing $T_{B}$ due to the temperature dependence of the resistance of the thin metallic films on the sapphire substrates. In the case of S2 and S3, for the purpose of data comparison, the $T_{B}$ depending residual resistances were subtracted in the data plot of following IVCs. On the other hand, the sample S1 has unknown components of the residual resistance which may be originated from the contact resistance between the SAM and the electrodes. The structure of the contact rigs shown in Figure 2a also may be related to this behavior. In this case, in order to compare the device characteristics depending on the applied bias voltages, we assumed a constant resistance at each bath temperature. For example, the $T_{B}$ depending IVCs shown in Figure 3(a-1) are plotted assuming three constant resistances of 52.1 Ω (20 K), 53.5 Ω (40 K), and 56.0 Ω (60 K), respectively.

Figure 3a displays the temperature dependence of the IVCs of three samples. The IVCs were obtained by increasing or decreasing of the applied bias voltage to the SAM as indicated by the arrows in Figure 3a. The IVCs of S1 at 20 K initially reach the critical current $I_{c}$ at about 30 mA. Then, the applied bias voltage to the sample increases with small voltage steps and reaches at around 5.3 V. After that the bias current increases up to ∼50 mA with increasing the applied bias voltage. Then, by decreasing the applied bias voltage, the IVC shows a hysteresis loop as seen in Figure 3(a-1).

The hysteresis loop of IVCs is observed in three samples and shrinks with increasing $T_{b}$. The maximum applied bias voltages at around 20 mA for three samples are ranging from 5 V to 6 V which is indicative of the heat-removal performance of this type of device structure. According to the previous studies for the IVCs of IJJs mesa structures, it is well known that the maximum applied bias voltage to the sample depends on the heating balance of the measurement system [68]. These are characterized by Joule heating caused by an applied current to the IJJs, a normal resistance of the IJJs, a cooling-system of measurement, the efficiency of the heat removal from the IJJs, etc.

The radiation intensity detected by the bolometer, $V_{\mathrm{out}}$, is plotted as a function of the applied bias voltage to the SAM in Figure 3b. It is noted that the scales of the vertical axes of Figure 3b are different each other. Three emitters mainly show the radiation at the return branch of the IVCs. In the return branch of the IVCs, it is generally well known that the number of the active Josephson junctions $N_{\mathrm{act}}$ may be switched as the applied bias voltages. The temperature dependencies of the radiation intensities are different from each other. From a simple comparison of the maximum radiation intensity among three samples, the thickest sample S3 shows the strongest radiation at 50 K.

In our previous studies [40,43], we evaluated the radiation intensity by using a commercial power meter (Erickson PM4, VDI) with comparing to the Si-composite bolometer and an InSb hot electron bolometer (HEB) detector (QFI/2BI, QMC Instruments). The radiation power detected by these bolometers can be estimated as *P* = 2$\sqrt{2}$$V_{\mathrm{out}}$/α, where α is a system optical responsivity. The system optical responsivities of the Si-composite bolometer and the HE bolometer are α = 11 [mv/nw] (AMP 200) and 3.3 [mv/nw] (AMP 1000), respectively [40]. The electromagnetic wave receiving area of the HEB is about four times larger than that of the Si-composite bolometer. In addition, in our previous study, we observed 6.5 μW when $V_{\mathrm{out}}$ of the HEB is $V_{\mathrm{out}}$∼2.5 V [43]. In order to estimate a total radiation power, we also need information of the radiation pattern and a detecting solid angle. With considering these relationships, the maximum $V_{\mathrm{out}}$ ∼ 800 mV (at AMP 200) of S3 at 50 K can be estimated very roughly to be few tens of microwatt.

**Figure 4:**
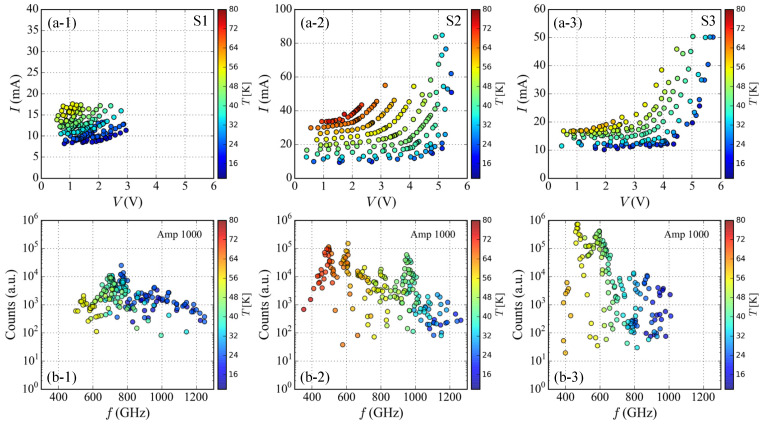
(**a**) Applied bias points on the IVCs for the radiation frequency measurement are displayed. The symbol colors correspond to the bath temperatures in the right bar codes. (**a-1**) sample S1; (**a-2**) samples S2; (**a-3**) samples S3. (**b**) Relationship between the observed radiation spectral peak frequencies and its intensities measured by the FT-IR spectrometer. The symbol colors also correspond to the bath temperatures in the right bar codes. (**b-1**) sample S1; (**b-2**) samples S2; (**b-3**) samples S3.

In order to clarify the radiation frequency characteristics on the IVCs more precisely, we measured radiation spectra by using the FT-IR spectrometer. The applied bias points on the IVCs for the spectral measurement are summarized in Figure 4a. The symbol colors correspond to $T_{b}$ in the right bar codes. As seen in Figure 4(a-1), in the case of sample S1, the radiations were obtained in low bias current and low voltage region only below 20 mA and 3 V. On the other hand, the radiations from the samples S2 and S3 were obtained at very wide region of the IVCs as seen in Figure 4(a-2,a-3). Unfortunately, at this moment, we do not have clear understanding about this difference.

The observed spectral peak frequencies and its intensities for the three SAMs are summarized in Figure 4b. The symbol colors also correspond to $T_{b}$ in the right bar codes. As mentioned in the temperature dependence of the IVCs, the hysteresis loop of IVCs strongly depends on $T_{b}$ and higher bias voltage can be applied to the IJJs at lower $T_{b}$. Therefore, as seen in Figure 4b, higher radiation frequencies can be obtained at lower $T_{b}$ and the observed radiation frequencies shift to lower frequencies with increasing $T_{b}$. This behavior is well understood from the temperature dependence of the IVCs and the ac Josephson effect.

The observed spectral peak intensities displayed in Figure 4b are plotted in logarithmic scale. From this plot, we can find clearly that there are several peaks enhanced in the frequency spectrum plot at characteristic frequencies. These enhancements can be understood from cavity modes determined by the size and shape of the SAMs. The resonance frequency of the lowest transverse magnetic mode of TM(1,0) can be expressed as $f_{1,0}$ = $c_{0}$/(2*nw*), where *w* is the width of the SAM, *n* is the refractive index, and $c_{0}$ is the speed of light in vacuum. According to previous studies [30,34,40,41], by using *n* = 4.2 and the width of the SAM, the resonance frequencies of three SAMs are estimated to be $f_{1,0}$ = 0.36 THz(S1), 0.45 THz(S2), and 0.60 THz(S3), respectively. The samples S2 and S3 show clear enhancement around these frequencies. In the case of the sample S2, spectral peak intensities are also enhanced at around 0.93 THz. It would be a higher cavity mode of TM(2,0). On the contrary to this, for the sample S1, there is no clear enhancement of the spectral peak intensity at around the frequency of the TM(1,0) mode. This would be related the temperature dependence of the IVCs and is discussed latter.

The spectral peak intensities observed around 0.5∼0.6 THz for the samples S2 and S3 seem to have two peaks. These peaks must be affected by the measurement condition of the data set. It means that an artificial effect due to the selection of measurement temperatures and bias points will be reflected. In addition, wire grids used in the FT-IR spectrometer may give an important effect for the spectrum intensity. In our spectrometer (FARIS-1), three wire grids are used in order to measure spectrum as an interferometer. We fixed them during the measurements for the samples. The direction of the wire grid placed at the electromagnetic wave entrance of the FI-IR spectrometer affects the observed spectral peak intensities because the transmittance of the wire grid depends on the polarization of the incident electromagnetic waves. In order to remove this kind of effect, we need a measurement data set with small temperature steps and small bias point steps from low to high $T_{b}$ with considering the radiation polarization. Therefore, we do not give further arguments on two-peak characteristics.

## 4. Discussion

Based on the results shown in Figure 4, three characteristic properties can be extracted and discussed more precisely below. The first pronounced feature is on the radiation intensity. According to the results seen in Figure 4b, the thickest sample S3 shows the strongest radiation among three SAMs. This behavior was actually pointed out from the early beginning [21], and it is expected to be proportional to $N_{\mathrm{act}}^{2}$. Therefore, the thicker mesa would be suitable for strong emission. The experimental result seen in Figure 4b seems to support the former behavior. This point will be discuss later more precisely by considering $N_{\mathrm{act}}$ estimated from the radiation frequency and the applied bias voltage to the IJJs.

The second feature is on the radiation frequency properties, which depend on the thickness of the SAMs. As seen in Figure 4a, the thinner sample S1 can generate higher radiation frequencies than the thicker sample S3 at low $T_{b}$. Since the radiation frequency is simply proportional to the voltage per junction, this result indicates that the applied bias voltage per junction of S1 at low $T_{b}$ is in fact higher than that of S3. This behavior is due to the reduction of the applied bias voltage per junction caused by Joule heating of the SAMs and is limited by the heat-removal performance determined by the structures.

The third feature is on the difference of the device working bath temperatures. In fact, this would be related to the critical current $I_{c}$ and the area of hysteresis loop of IVCs. As seen in Figure 2a, the sample S2 shows higher $I_{c}$ and wider area of hysteresis loop of IVCs above 60 K than those of samples S1 and S3. This difference is clearly reflected in the temperature dependence of the observed radiation frequencies as seen in Figure 4b. The sample S2 works up to higher $T_{b}$ than the samples S1 and S3. The samples S1 and S3 show no clear emission above 65 K, however, the sample S2 shows clear emission up to around 80 K as seen in Figure 4(b-2). This result suggests that $I_{c}$ and the area of the hysteresis loop of IVCs have an important role for the device working bath temperature.

In principle, the critical current $I_{c}$ is originated from the sample geometries as well as the material characteristics of Bi2212 crystals. According to a simple comparison of the cross-sectional area of the samples, the relationship of $I_{c}^{S1}$ > $I_{c}^{S2}$ > $I_{c}^{S3}$ is expected. However, the experimental results displayed in Figure 3a show the relationship of $I_{c}^{S2}$ > $I_{c}^{S1}$ > $I_{c}^{S3}$. The highest $I_{c}$ of S2 can be understood from the highest $T_{c}$ of the sample. It is known that $T_{c}$ and $I_{c}$ become smaller with decreasing the carrier doping level of the crystals. In addition, in the previous works for higher bath temperature operation of Bi2212-THz emitters [38,67], the mesa structures made of Bi2212 single crystals with $T_{c}$ ∼ 90 K were used. They observed clear emission and the wide area of hysteresis loop of IVCs with large $I_{c}$ at around 80 K. Our observed results discussed here are consistent with such studies.

Consequently, we can control the radiation frequency, the device working bath temperature, and the radiation intensity by considering the above characteristic properties. For example, the device condition of sample S2 is suitable for higher working bath temperature with strong emission at TM(1,0) as well as for higher frequency generation at lower $T_{b}$. It is noted that the above characteristics strongly depend on the heat-removal performance of the device. It would be obtained from our proposed structures as displayed in Figure 2.

**Figure 5:**
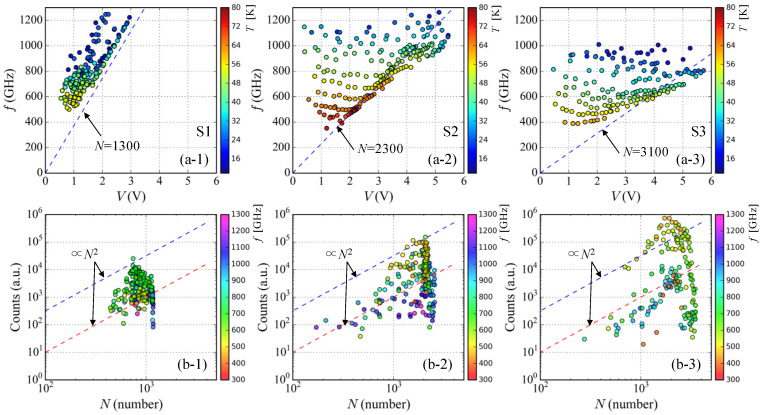
(**a**) Relationship between the applied bias voltages and the observed radiation frequencies for the three SAMs. The dashed lines indicate the relationship between the radiation frequency and the applied bias voltage to the SAMs calculated from the ac-Josephson effect and thicknesses of the SAMs. The symbol colors correspond to the bath temperatures in the right bar codes. (**a-1**) sample S1; (**a-2**) samples S2; (**a-3**) samples S3. (**b**) Relationship between $N_{\mathrm{act}}$ and the spectral peak intensities. In this case, the symbol colors correspond to the observed radiation frequencies in the right bar codes. (**b-1**) sample S1; (**b-2**) samples S2; (**b-3**) samples S3.

Lastly, we discuss the radiation frequency and intensity characteristics by considering the multi branching characteristics of IVCs. Figure 5a shows a relationship between the applied bias voltages to the SAM and the observed radiation frequencies. The symbol colors correspond to $T_{b}$ in the right bar codes. Note that the same data shown in Figure 4 are used for these data plots. As mentioned above, the higher frequencies are observed at lower $T_{b}$ and the radiation frequencies shift to lower frequency side with increasing $T_{b}$. This $T_{b}$-dependence of the radiation frequency characteristics are clearly indicated in Figure 5a.

The blue dashed lines in Figure 5a denote the relationship between the radiation frequency and the applied bias voltage to the SAMs calculated from the ac Josephson effect and the thicknesses of the SAMs. The blue dashed lines also indicate that the whole of the IJJs in the SAMs is in the resistive state. Thus, the data points plotted in the upper left side of the blue dashed line originated from the radiation in the multi branching structure of IVCs as seen in Figure 3. It is clear that the observed radiation frequency is proportional to the applied bias voltage to the SAMs according to the ac Josephson effect. The data points deviated from the blue dashed line above 4 V for the samples S2 and S3 are attributed to the Joule heating because that kind of deviation for the thinner sample S1 is very small. It is also noted that according to experimental results by using four electrical terminals on a top surface of rectangular mesa device [70], the inhomogeneous local temperature distributions in the ab-plane of the mesa device caused by a contact resistance prevent from obtaining proper value of the applied bias voltages because of appearance of a potential distribution at the top electrode.

According to the ac Josephson effect, the radiation frequency of the emitter, $f_{\mathrm{ob}}$, can be expressed as $f_{\mathrm{ob}}$ = ($\frac{2e}{h}$)($\frac{1}{N_{\mathrm{act}}}$)$V_{\mathrm{app}}$, where $V_{\mathrm{app}}$ is the voltage applied across the IJJs, *e* and *h* are the electric charge and Planck constant, respectively. Therefore, by using the experimental data of $f_{\mathrm{ob}}$ and $V_{\mathrm{app}}$, $N_{\mathrm{act}}$ can be estimated to be as $N_{\mathrm{act}}$ = ($\frac{2e}{h}$)($\frac{1}{f_{\mathrm{ob}}}$)$V_{\mathrm{app}}$. The relationship between $N_{\mathrm{act}}$ and the observed spectral peak intensities for the three SAMs are displayed in Figure 5b. In this plot, the symbol colors correspond to the observed radiation frequencies in the right bar codes. It is clear that the spectral peak intensities increase with increasing $N_{\mathrm{act}}$. The radiation intensity is expected to be proportional to $N_{\mathrm{act}}^{2}$ according to the previous study [21]. Thus, the arbitrary plots of the $N^{2}$-relationship with two different offsets are also displayed by the dashed-lines in Figure 5b. Some data points seem to follow the $N^{2}$-like-relationship, however, it is not adequate to mention clear relationship between $N_{\mathrm{act}}$ and the spectral peak intensities at this moment. In order to understand this relationship more precisely, we need additional experiments and data analysis. This is one of our future subjects.

Finally, we mention an additional feature for the radiation intensity related to $N_{\mathrm{act}}$. The samples S2 and S3 show the maximum peak intensity at around $N_{\mathrm{act}}$ ∼ 2000; however, the maximum spectral peak intensity of the sample S3 is much higher than that of the sample S2 as seen in Figure 5(b-2,b-3). This property also can be confirmed in Figure 3. This result suggests that the thicker sample S3 has higher emission efficiency than the sample S2. According to the patch antenna theory [77,78], it is known that the radiation efficiency of the patch antenna depends on the thickness of the patch and a thicker patch antenna shows higher radiation efficiency. However, there is a limitation for the patch thickness in order to stabilize the resonance condition. The experimental result seen in S2 and S3 seems to suggest the existence of this kind of thickness effect. The detail understanding of this point is very important to improve the radiation power and is our future subjects.

## 5. Conclusions

In this study, we have compared the radiation characteristics of the three rectangular SAMs made of different thicknesses of Bi2212 crystals. The radiation intensity, the radiation frequency, and the device working bath temperature characteristics are well understood on the basis of the difference of the three samples. The stronger radiation intensity is observed from the thickest SAM. This feature is discussed trough the $N^{2}$-relationship and the radiation efficiency of the patch antenna. The thinner SAM can generate higher radiation frequencies than the thicker one due to the difference of the applied voltage per junctions limited by the heat-removal performance of the device structure. The device working bath temperature seems to be related to the material characteristics of Bi2212 crystals used for the SAMs as reflected in $T_{c}$, $I_{c}$, and the area of hysteresis loop of IVCs. Some of the observed results are required additional research by using well size controlled SAMs. Nevertheless, the observed characteristic features in this study are very useful to design a Bi2212-THz emitter with desired emission characteristics for many applications.
